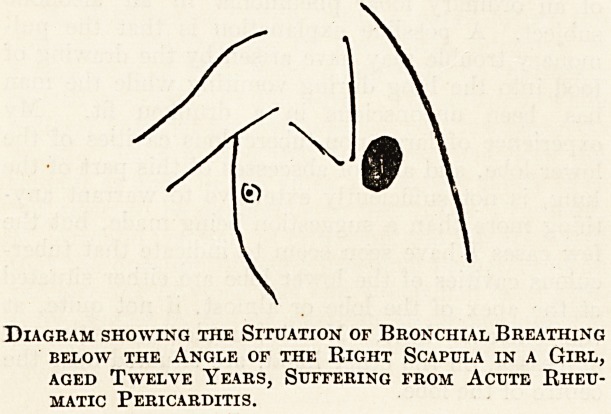# Some Points on Various Affections of the Lungs.—II

**Published:** 1911-05-20

**Authors:** Theodore Fisher


### Hospital Clinics.

### SOME POINTS ON VARIOUS AFFECTIONS OF THE LUNGS.—II.

By THEODORE FISHER, M.D., F.R.C.P.

It has been previously mentioned that calcareous nodules are not uncommonly met with in the lower lobe, and it was suggested that these nodules need not necessarily be of tuberculous origin. Cavities sometimes of large size may be met with in the lower lobe, and these may obviously be of non-tuberculous origin. Yet more often such cavities are undoubtedly tuberculous. There may, for example, be a cavity in the lower lobe and chronic tuberculosis without cavitation of the upper lobe, or, as in a case of a man aged forty-four, on whom I made an autopsy, there may be a large cavity in one lower lobe, and the upper lobe on the same side may be quite healthy, while comparatively slight tuberculous disease of the upper lobe may be present on the opposite side. Curiously enough, on more than one occasion I have seen fatal hæmoptysis arising within a cavity in the lower lobe.

### Some Interesting Cases.

Brief notes of two of these cases may be of interest. In one case a man, aged forty-one, a fortnight before admission into a hospital coughed up half a pint of blood. Two days later he coughed up eight ounces, and smaller quantities on two succeeding days. He eventually died of hæmoptysis a week later. After death there was found a cavity the size of a plover's egg at the extreme base of the left lung. This cavity was almost completely filled by a false aneurysm; that is to say, the hæmorrhage of three weeks before death had resulted in the formation of firm layers of clot which lined the walls of the cavity. The hæmorrhage, which ended fatally, had burst through this firm clot and had partly separated it from the wall of the cavity in the lung. Tuberculosis in this case was not limited to the lower lobe. There was a smaller old cavity in the upper lobe, and fibrosis around. Some comparatively recent tuberculous disease was present in the lower lobe.

Another case occurred in a woman aged thirty. There also the cavity, which was the size of a medium-sized marble, was at the extreme base of the lower lobe, in this instance, of the right lung. Here, too, an aneurysm filled the cavity, but it was a true aneurysm, and a branch of the pulmonary artery could be traced entering it. This aneurysm burst into a bronchial tube and death had occurred from sudden and profuse hæmoptysis. In this case there was no cavitation of the upper lobe, but slight old fibrosis of the upper lobe of the opposite lung was present. In both of these cases there was definite evidence of the tuberculous origin of the cavities, yet I have met with a fatal case of hæmoptysis from a cavity in the lower lobe in which there was no evidence whatever of the presence of tubercle. This occurred in a man aged thirty. In the centre of the right lower lobe was a large cavity the size of a tangerine orange. This was full of blood-clot, the man having died from fatal hæmoptysis.

### Non-Tuberculous Cavitation.

The origin of these non-tuberculous cavities in the lower lobe is obscure. Twice in alcoholic subjects I have made a post-mortem examination in which an abscess cavity with foul-smelling contents has been found in the centre of a lower lobe. The cavity has been of large size with a well-defined wall. The lung substance immediately around has been only partially consolidated, and has borne no resemblance to true pneumonia, the lung nowhere being really solid. It is obvious that such a cavity has not arisen by the breaking down of an ordinary lobar pneumonia in an alcoholic subject. A possible explanation is that the pulmonary trouble may have arisen by the drawing of food into the lung during vomiting while the man has been unconscious in a drunken fit. My experience of large non-tuberculous cavities of the lower lobe, and also of abscesses of this part of the lung, is not sufficiently extensive to warrant anything more than a suggestion being made, but the few cases I have seen seem to indicate that tuberculous cavities of the lower lobe are either situated at the apex of the lobe or almost, if not quite, at the extreme base. Non-tuberculous cavities or abscesses, on the other hand, are situated near the centre of the lobe.

### Diagnosis of the Lesion.

To digress again from the appearance of morbid anatomy to the experience of the bedside, it is worthy of note that it is possible to diagnose a cavity of the lower lobe when no such cavity exists. Several times a surgeon, with the concurrence, no doubt generally of a physician, has opened the chest expecting to find a cavity in the lower lobe, only to meet with disappointment, and not uncommonly to hasten the end of the patient. In the post-mortem room is generally found some general dilatation of the bronchial tubes in the lower lobe, perhaps of the whole lung. Aware of this possible error of diagnosis I once warned a surgeon as to what he might find. My warning did not prevent the operation, and I had an opportunity later of seeing the lung, which contained throughout dilated bronchial tubes. The most common situation for the loud bronchial or even amphoric breathing which leads to the diagnosis of a cavity to be heard is just below and internal to th[illegible] inferior angle of the scapula when the arms are in front of the chest. The reason for the presence of intensified abnormal physical signs at this spot is not clear. It seems to me, however, that any abnormal condition which leads to conduction of tracheal sounds to the lower lobe may cause them to be best heard near the lower angle of the scapula, provided that the abnormal condition does not reach the surface of the lung. Thus a patch of considerable size of consolidation of the lung should it be present deep within the lung may lead to the presence of bronchial breathing at this spot only.

### Bronchial Breathing.

Another condition which leads to bronchial breathing being heard at this spot is a rapidly produced enlargement of the heart, such as occurs in rheumatic pericarditis in children. The reason for the appearance of bronchial breathing below the inferior angle of the scapula is especially obscure in this condition of enlargement of the heart. To leave this special cause, however, for the moment, and consider why bronchial breathing should be heard in any condition, it may be mentioned that it seems possible that its presence at one particular spot may be due to the shape of the chest. Tracheal sounds conducted to the lower lobe must radiate in all directions through the lobe and the lung. They will strike the whole of the inner surface of

Diagram showing the Situation of Bronchial Breathing below the Angle of the Right Scapula in a Girl, aged Twelve Years, Suffering from Acute Rheumatic Pericarditis.

the chest. A considerable portion of the waves of sound will be reflected back from this surface of the chest, and there may be a point to which these reflected waves converge, and this converging point may be the spot which has been indicated near the inferior angle of the scapula.

### Consolidation and Bronchial Breathing.

Although the presence of bronchial breathing occurring in association with the large heart of acute pericarditis is possibly as much a question to be considered in discussing matters bearing upon the heart as upon the lungs, a brief consideration of it here may not be out of place. The enlarged heart by pressing upwards on some of the divisions of the bronchus to the lower lobe, occasionally produces a strip of collapse on the lower lobe. I used to think that this strip of collapse must be in some way responsible for the appearance of the bronchial breathing. One case, however, drove this idea from my mind. I entered a hospital ward when a physician was examining a case of acute enlargement of the heart associated with pericarditis in a child. The physician and the hospital resident with him heard bronchial breathing over the right side of the chest behind, and considered that pneumonic consolidation was present. At the time it did not occur to me that this was one of those cases in which bronchial breathing may be present without any marked abnormal condition of the lungs, and I did not ask permission to listen to the chest. This I much regretted on the following day, when I had an opportunity of examining the heart and lungs after death. The right lower lobe proved to be free from any abnormal condition which could be detected with the naked eye. There was not even the strip of collapse sometimes seen. This case much puzzled me, and I can only think that the large heart itself, as it projects backwards, compressing and displacing lung substance, acts as the conductor of the tracheal sounds, and that these sounds are best heard at the inferior angle of the scapula for the reasons above given. There is one difficulty which occurs to me, however, in accepting this view. It is that it is difficult to understand why the sounds should not be present in chronic as well as in acute enlargement of the heart. Perhaps they are sometimes. However that may be, there is no question with regard to the occasional appearance of marked bronchial breathing at the inferior angle of the scapula in some cases of rapid enlargement of the heart associated with acute rheumatic pericarditis in children.

### An Illustrative Case.

Above is a copy of a diagram in my notes taken from such a case. The area of bronchial breathing was present in a girl aged twelve, suffering from acute rheumatic pericarditis with great enlargement of the heart. It may be of interest possibly to mention that the note was made in July 1894, before I had become aware that bronchial breathing is noted occasionally in this situation in cases of rheumatic pericarditis. The bronchial breathing remained for nearly a month, but had disappeared when the girl left the hospital. A year later she returned, and died in a few days. The heart at the time of death was greatly enlarged; it weighed 14 ounces.

It should be mentioned that in non-tuberculous cavities of the lower lobe were not included dilated bronchial tubes of a size and shape that deserve the name of cavities. Some reference to varieties of dilatation of the bronchial tubes may be made later.

## Figures and Tables

**Figure f1:**